# Enhanced recovery of alkaline protease from fish viscera by phase partitioning and its application

**DOI:** 10.1186/1752-153X-7-79

**Published:** 2013-04-30

**Authors:** Sunantha Ketnawa, Soottawat Benjakul, Tau Chuan Ling, Oscar Martínez-Alvarez, Saroat Rawdkuen

**Affiliations:** 1 Program of Food Technology, School of Agro-Industry, Mae Fah Luang University, Chiang Rai 57100, Thailand; 2 Department of Food Technology, Faculty of Agro-Industry, Prince of Songkla University, Hat Yai, Songkhla 90112, Thailand; 3 Institute of Biological Sciences, Faculty of Science, University of Malaya, Kuala Lumpur 50603, Malaysia; 4 Institute of Food Science, Technology and Nutrition (ICTAN-CSIC), Spanish National Research Council, José Antonio Novais 10, Madrid 28040, Spain

## Abstract

**Background:**

Too many different protein and enzyme purification techniques have been reported, especially, chromatographic techniques. Apart from low recovery, these multi-step methods are complicated, time consuming, high operating cost. So, alternative beneficially methods are still required. Since, the outstanding advantages of aqueous two phase system (ATPS) such as simple, low cost, high recovery and scalable, ATPS have been used to purify various enzymes. To improve purification efficiency, parameters affected to enzyme recovery or purity was investigated. The objectives of the present study were to optimize of alkaline protease recovery from giant catfish fish viscera by using ATPS and to study of hydrolytic patterns against gelatin.

**Results:**

Using 70% (w/w) crude enzyme extract (CE) in system (15% PEG2000-15% sodium citrate) provided the highest recovery, PF and K_E_. At unmodified pH (8.5) gave the best recovery and PF with compare to other pHs of the system. The addition of 1% (w/w) NaCl showed the recovery (64.18%), 3.33-fold and 15.09 of K_E_ compared to the system without NaCl. After addition of 10% (w/w) sodium citrate in the second ATPS cycle, the highest protease recovery (365.53%) and PF (11.60-fold) were obtained. Thus, the top phase from the system was subjected to further studied. The protein bands with molecular weights (MWs) of 20, 24, 27, 36, 94 and 130 kDa appeared on the protein stained gel and also exhibited clear zone on casein-substrate gel electrophoresis. The β, α_1_, α_2_ of skin gelatin extensively degraded into small molecules when treated with 10 units of the extracted alkaline protease compared to those of the level of 0.21 units of Flavourzyme.

**Conclusions:**

Repetitive ATPS is the alternative strategy to increase both recovery and purity of the alkaline protease from farmed giant catfish viscera. Extracted alkaline protease exposed very high effectiveness in gelatin hydrolysis. It is suggested that the alkaline protease from this fish viscera can further be used in protein hydrolysate production.

## Background

Nowadays, the efficient and economical downstream processes for the partitioning and purification of biomolecules that give high recovery and high purity of the product are required by industries [1]. Recovery and purification of biomolecules is a complicated, cost intensive process and can account for up to 70% of the production cost of biomolecules [2]. Most previous works reported that to purify protease from fish digestive organs involved several methods, including ammonium sulphate precipitation [3], size exclusion chromatography [4], ion-exchange chromatography [5], hydrophobic interaction chromatography and affinity chromatography [6,7]. These multi-step purification methods result in very high cost of operation, difficult to operate and scale up, time consuming purification process and relatively low recovery.

Aqueous two phase system (ATPS) could be an efficient method for the recovery of protease due to the ease and lower cost [8]. ATPS have several advantages in comparison with conventional methods for the isolation and purification of proteins such as low cost, nontoxic, the possibility of application on a large scale and the short time required for reaching equilibrium [9]. ATPS is a very mild method of protein purification, and denaturation or loss of biological activity is not usually seen [10].

One of the critical factors for enzyme purification by using ATPS is the selection of the appropriate system conditions. The selective distribution of ATPS constituents may be affected by different factors for example the nature and size of the bioactive compounds, initial composition of the system, molecular structure and chain size of the polymer, type of salt, system temperature, pH, NaCl addition, and number of cycle of ATPS [10,11]. The pH value and the presence of electrolytes in the system have a pronounced effect on the partitioning of proteins between the two phases [12].

The Giant catfish (*Pangasianodon gigas*) is one of the economically important farmed fish that has been successfully cultured in Northern Thailand, especially in Chiang Rai province [13]. The edible portion of farmed giant catfish is about 50-60%, implicating that another 40-50% of whole weight generated as a by-product [14]. Its viscera accounts for about 5-10% of the entire weight [15,16]. Besides, the discarding of fish by-products creates the environmental problem as well as disposal problem due to high fat content and other proteins that boosting microorganism growth.

Viscera have wide biotechnological potential as a source of digestive enzymes that may have some unique properties for industrial applications, e.g. in the detergent, food, pharmaceutical, leather and silk industries [17]. The most important of these are acid stomach enzymes, pepsin, and alkaline intestine enzymes. The main alkaline protease in fish viscera are trypsin, chymotrypsin and elastase, all belonging to the serine-protease family (E.C. 3.1.21.x) [18]. The use of alkaline proteases has increased remarkably, since they are both stable and active under harsh conditions, such as at temperatures of 50-60°C, high pH and in the presence of surfactants or oxidizing agents [19].

The alkaline protease was extracted from the viscera (intestine) of Nile tilapia by using heat treatment, ammonium sulphate fractionation and Sephadex G-75 gel filtration, presenting yield and purification of 30% and 22-fold, respectively [20]. Klomklao et al. [8] reported partitioning of spleen proteinase from yellowfin tuna by ATPS (comprising PEG1000 (15%, w/w) and magnesium sulfate (20%, w/w)) showed high yield (69.0%), and purification fold (6.61). Various related works reported about extraction and purification of enzymes from fish viscera and their application in different aspects [15,20,21]. Nonetheless, there are few practically up-to-date reports related to enhance of recovery and purity of enzymes from fish viscera. Hence, the objectives of present study were to enhance alkaline protease recovery by using farmed giant catfish viscera as raw material in ATPS, to study different parameters influenced on partition of enzymes, and to apply extracted enzymes in gelatin hydrolysis.

From previous researches, fish viscera can be valuable sources for enzyme extraction. Furthermore, the extracted enzymes from viscera are distinctively useful in industrial applications. Thus, study on the potential use of viscera from farmed giant catfish is needed. Until now, no information has been reported on the optimization of alkaline protease from this fish. Nevertheless, no information regarding application of alkaline protease on hydrolysis of farmed giant catfish skin gelatin.

## Results and discussion

### Effect of crude enzyme extracts on the protease partitioning

**Figure 1:**
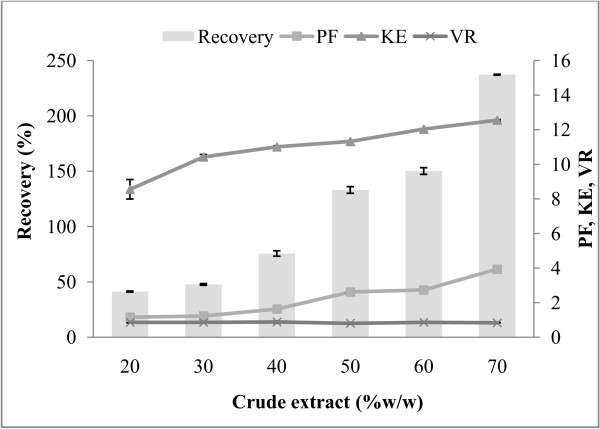
**Influence of crude enzyme extract on protease recovery (%), purification factor (PF), partition coefficient (K**_**E**_**), volume ratio (V**_**R**_**) of alkaline protease partitioning in 15% PEG 2000-15% sodium citrate, pH 8.5.**

The partition experiments were carried out at different amounts of CE (20, 30, 40, 50, 60, and 70%, w/w ATPS). The loaded CE in the enzyme partitioning had played a major role in ATPS. An increase in amount of CE, positively affected the recovery of enzyme in a large feed volume using ATPS [22]. The effect of CE from 20-70% (w/w) on alkaline protease partitioning is shown in Figure 1. It illustrated that the K_E_, recovery, and PF of extracted protease were gradually increased for six different amounts of CE manipulated. The maximum K_E_ (12.55), recovery (157.13%) and PF (3.94) was exhibited at the CE content of 70% (w/w). The more CE used, the more partition coefficient of protease (K_E_) gained. K_E_ ranged from 8.55 (20%, w/w) to 12.55 (70%, w/w) indicating that more alkaline protease partition to the top phase. For volume ratio (V_R_), it ranged from 0.74 (70%, w/w) to 0.87 (20%, w/w). The V_R_ were converse with the K_E_ when increasing amount of CE. When CE contents were increased, solution detached from polymer rich phase to salt rich phase. Increasing loaded mass into the ATPS may decrease the phase V_R_ and thus alter the composition of the particular system [23,24]. Larger amounts (>20%, w/w) of CE in the ATPS affect the composition/properties of the ATPS and decrease the V_R_. Protein concentration has an important effect on the partitioning of proteins in ATPS. This depends on the higher levels of solubility of the protein in each phase. Hence, the partitioning observed at low protein concentrations can be very different to that observed at high concentrations [10]. However, from previous reports, the K_E_ and V_R_ are decreased when a higher amount of the sample is loaded and thus the system is not at optimum condition. From the report of Ng et al. [23] showed that, use only 20% (w/w) of crude load provided the highest recovery (70.53%) and PF (13.1) in partitioning cyclodextrin glycosyltransferase derived from *Bacillus cereus* from rotten potato by ATPS. The previous study of Amid et al. [22] on the partitioning of serine protease from mango peel using an alcohol/salt ATPS found that 20% (w/w) crude load indicated maximum capacity on the basis of 10 g of ATPS with the yield of 95.8% and 11-fold of purity.

### Effect of pH on the protease partitioning

**Figure 2:**
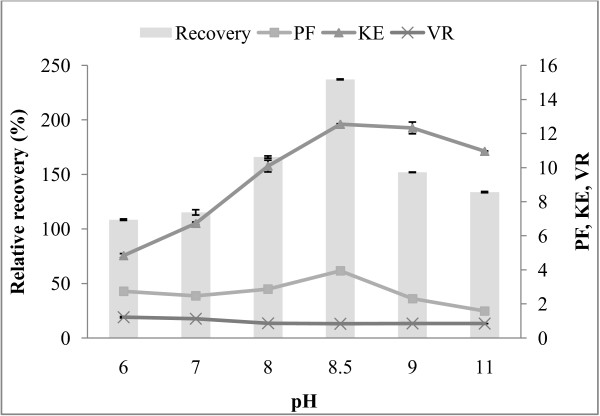
**Influence of pH on protease recovery (%), purification factor (PF), partition coefficient (K**_**E**_**), volume ratio (V**_**R**_**) of alkaline protease partitioning in 15% PEG 2000-15% sodium citrate containing 70% (w/w) crude enzyme extract.**

The pH of the ATPS affects the partitioning because it may alter the charge of the solute or it may alter the ratio of the charged molecules. The net charge of the protein depends on whether the pH is greater than pI (negative), lesser than pI (positive), or equal to pI (zero). Several researchers reported that at higher pH, the negatively charged bio-molecule prefers the top phase and partition coefficient increase. It may be because of the electrostatic interactions between the biomolecule and PEG units. Moreover, the change in pH affects the phase composition which in turn affects the partitioning behaviour [25]. The influence of pH on the partitioning of protease from fish viscera was investigated by using the ATPS composition of 70% (w/w) CE and 15% PEG2000-15% sodium citrate which gave the highest protease recovery. The experiments were carried out at different pH values (6, 7, 8, 9, 10, and 11) in comparison to the pH 8.5 of the control system (without pH adjustment). Effect of pH on recovery, PF, K_E_, and V_R_ of alkaline protease in the PEG-rich top phase compared to those values of the top fraction of previous step was depicted in Figure 2. The recovery increased from 115.98 to 127.03%, PF 2.74 to 2.87, K_E_ 4.84 to 10.09 (pH 6 to pH 8). At low pH, the target protease is positively charged and prefers the salt-rich phase. This explains the reduction of both K_E_ and enzyme recovery in the experiment at pH 6–7. The maximum recovery of 127.03% and purification factor of 2.87 were observed at pH 8. It was found that increasing the pH from 6 to 11 markedly increased in the K_E_ 6.74 to 12.33. The increase in pH augments the negative charge of the protein surface above the isoelectric point. The negatively charged protein concentrates in the top phase, and the partition coefficient therefore increases [2]. At alkaline condition (pH 11.0), decrease of the recovery (100.40%) was found when compared to that of unmodified pH. The change in K_E_ can be explained by considering the change in the net charge of the enzyme surface compared to their isoelectric points [2]. A decrease in the recovery activity and PF of alkaline protease was observed at the pH below and above 8. As the pH of the system is increased above the isoelectric point, the alkaline protease surface charge becomes negative. As a result, partitioning of the enzyme into the salt-rich bottom phase decreased [10]. Higher pH than 8.0 shows a significantly negative recovery and purity of the enzyme, probably due to protein denaturation and precipitation at high pH. It might be concluded that increase in the K_E_, PF and protease recovery at pH 8.0 was the result from the protease become closely to its neutral charge. At high pH (9.0-11.0), the recovery and PF are lowered compared to pH (8.0). This is probably due to in appropriate state of the charge in enzyme active site at the more alkaline condition**.** A similar observation was reported by Oliveira et al. [26] working on the effect of pH on partitioning of commercial trypsin in the phase systems containing PEG-cashew-nut tree gum ATPS. The results showed that the change of pH from the 6.0 to 7.0 increases K_E_ and from 7.0 to 8.0, decreases K_E_. The highest recovery (93.6%) and purification factors (11.6) were obtained at pH 8 on the study of the partitioning of serine protease from mango peel using an alcohol/salt ATPS [22].

### Effect of NaCl on the protease partitioning

**Figure 3:**
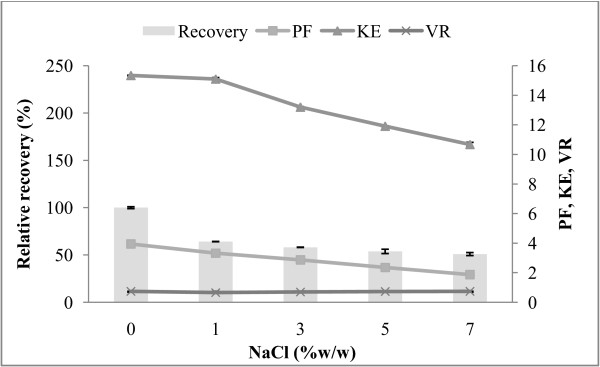
**Influence of NaCl addition on protease recovery (%), purification factor (PF), partition coefficient (K**_**E**_**), volume ratio (V**_**R**_**) of alkaline protease partitioning in 15% PEG2000-15% sodium citrate containing 70% (w/w) crude enzyme extract, pH 8.5.**

Partitioning of protease from alkaline protease in the presence of NaCl was also studied in the system of 70% (w/w) CE and 15% PEG2000-15% sodium citrate. The effect of NaCl concentration on the enzyme partitioning is shown in Figure 3. The presence of NaCl in ATPS alters partition coefficient because of the differential distribution of the salt ions between the phases. The hydrophobic ions force the partitioning of their counter ions to the more hydrophobic phase and *vice versa*[25]. From the result, NaCl concentration (1-7%, w/w) in the PEG-sodium citrate system had adverse effect on alkaline protease partition. The result showed that K_E_ decreased with the addition of NaCl. The K_E_ of alkaline protease decreased from 15.34 (no NaCl addition) to 10.67 (7%, w/w NaCl). V_R_ increased from 0.67 (1%, w/w) to 0.74 (7%, w/w). The maximum recovery of alkaline protease in the present study was 64.18% with PF 3.33-fold at 1% (w/w) NaCl addition. The recovery has practically decreased with the variation in salt concentration, indicating that the phase diagram is not influenced significantly by the variation in salt concentration. Occurrence of this effect may be due to a decrease in protein solubility as a result of the addition of NaCl [2]. Higher concentration of NaCl shows a significantly negative effect on partitioning and yield of the enzyme, probably due to protein denaturation and precipitation at high concentration of this salt. Besides, it can be presumable that the hydrophobicity of alkaline protease was lower than those of other proteins composed in the CE. As a consequence, the protease was more transferred to the bottom phase of salt. These results were in agreement with Amid et al. [22] who studied the K_E_ and recovery of serine protease from mango peel in 16% (w/w) of 2-propanol and 19% (w/w) of potassium phosphate at pH 8.0 ATPS, using NaCl at different concentrations, ranging from 1% (w/w) to 10% (w/w). The K_E_ and recovery of serine protease were significantly decreased from 64.5 and 96.7 at 5% (w/w) NaCl to 30.2 and 51.3 at 7% (w/w) NaCl, respectively.

According to the results, the ATPS formed 70% (w/w) of CE with 15% PEG2000-15% sodium citrate systems, without pH modification and NaCl addition, was chosen for the subsequent studies.

### Repetitive ATPS for protease recovery

**Figure 4:**
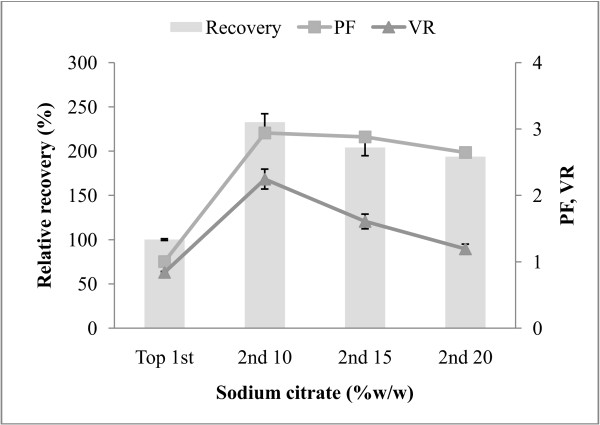
**Influence of sodium citrate addition in the second ATPS on protease recovery (%), purification factor (PF), volume ratio (V**_**R**_**) of alkaline protease partitioning in 15% PEG2000-15% sodium citrate containing 70% (w/w) crude enzyme extract, pH 8.5.**

The maximum alkaline protease recovery from the first ATPS was used as the starting material for the second ATPS. To increase the protease recovery and purity, additional sodium citrate 10, 15 and 20% (w/w) was added to the first cycle top fraction from 70% (w/w) CE with 15% PEG2000-15% sodium citrate ATPS. The reason for carrying out the second cycle of ATPS was that sometimes the first ATPS cycle could not remove the contaminant proteins efficiently. The highest protease activity recovery (365.53%) and purity (11.60-fold) was found in the top phase after additional 10% (w/w) sodium citrate was added (Figure 4). From the second ATPS, the overall result of protease activity recovery, as well as of final purity, is extensively better than the first ATPS (157.13% and 3.95-fold) (Table 1). At 10% (w/w) salt content, nearly three times protease recovery and purity were recovered in the second top phase compared with that presented in the first top phase of ATPS. A further increase of sodium citrate concentration could reach a saturation point resulting in un-dissolve of the salt. It can be pointed that the second cycle should be needed for increasing both recovery and PF of enzyme.

**Table 1:** Two steps of aqueous two phase partitioning of alkaline protease from farmed giant catfish viscera

**Step**	**Phase**	**Total activity (U)**	**Total protein (mg)**	**Specific activity (U/mg)**	**Purification (fold)**	**Recovery (%)**
Crude extract^a^	-	2,916.67±11.00^b^	8.32±0.05	350.51±3.57	1.00	100
1st ATPS^c^	Top	4,582.91±45.72	3.31±0.07	1,385.73±16.49	3.94±0.03	157.13±1.57
2nd ATPS^d^	10	10,965.60±10.18	2.70±0.01	4,064.79±5.99	11.60±0.02	365.53±15.11

^a^ crude enzyme extract from farmed giant catfish viscera (1:5 (w/v) of the minced viscera to 10mM Tris–HCl buffer with 10 mM CaCl_2_, pH 8.0).

^b^ values are given as mean ± S.D. from triplicate determinations.

^c^ 1st ATPS consists of 15% PEG2000-15%sodium citrate containing 70% (w/w) crude enzyme extract.

^d^ 2nd ATPS consists of the top phase of the second cycle ATPS, additional 10% (w/w) sodium citrate.

### Protein pattern and zymography

**Figure 5:**
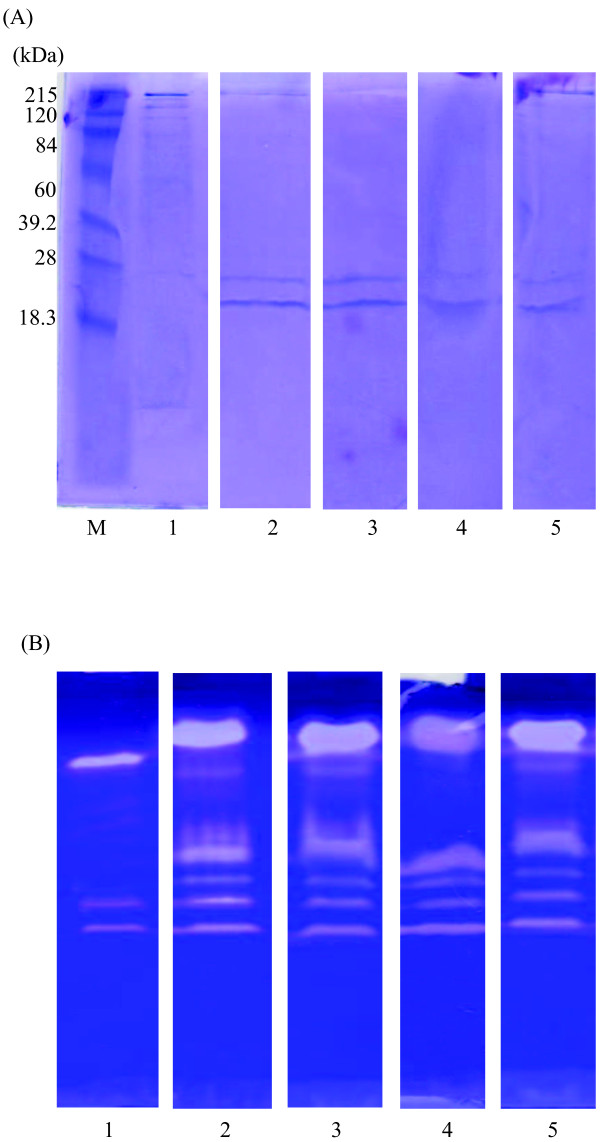
**SDS-PAGE patterns (5A) and activity staining (5B) performed using 4% stacking gel and 15% separating gel of alkaline protease from farmed giant catfish viscera 15% PEG2000-15% sodium citrate ATPS.** [8 and 2 μg protein were loaded into the gel for protein patterns and activity staining, respectively.] Lane 1: crude enzyme extract; 2: top phase of effect CE; 3: top phase of effect pH; 4: top phase of effect NaCl addition; 5: top phase of repetitive ATPS; M: molecular weight protein makers.

Protein patterns of the CE and their fractions from ATPS are shown in Figure 5A. CE contained a variety of proteins with different molecular weights (MW). The major protein bands with the MWs of 215, 130 and 94 kDa were found in the CE. Smear protein band with MW of 24 kDa was observed in CE. Besides, the protein bands with MWs of 20, 24, and 215 kDa of alkaline protease appeared. Klomklao et al. [5] reported that the MW of purified trypsin from the viscera of hybrid catfish by size exclusion chromatography and SDS-PAGE was 24 kDa. The MW of partial purified alkaline protease (Trypsin-like enzyme) from farmed giant catfish viscera was estimated 24 kDa when compared with trypsin from hybrid catfish viscera [5]. MW of trypsin was 24 kDa [18]. Rawdkuen [15] revealed that MW of trypsin recovered from the viscera of farmed giant catfish by three-phase partitioning was approximately 25 kDa.

Activity staining of ATPS fractions by using casein as substrate was shown in Figure 5B. A protein bands with the MWs of 20, 24, 27, 36, 94 and 130 kDa were estimated from the activity bands on the casein substrate gel electrophoresis. The presence of the clear zone suggested that it is the protease that can hydrolyze casein in the gel. There were 3 major clear zones (24, 36 and 130 kDa) that can be distinctively observed. The apparent MWs of trypsin-like enzyme were estimated to be 48, 23 and 23 kDa for skipjack, tongol and yellowfin tuna spleen, respectively [8]. MWs of trypsin-like enzymes from pyloric caeca brownstripe red snapper were 20, 24–29, 45 and 97 kDa; bigeye snapper were 17, 20, 22, 45 and 97 kDa; and threadfin bream were 20, 22, 36 and 45 kDa [27]. Slightly greater band intensity at 24–36 kDa in ATPS fraction was observed, suggesting the higher specific activity of alkaline protease loaded into the gel and supplied interesting results for further purification. These 24–36 kDa fractions also contained most of the interfering proteins. Consequencely, the extracted protease should demonstrate to further purification step namely size exclusion chromatography for gaining a single band of the enzyme.

### Gelatin hydrolysis study

**Figure 6:**
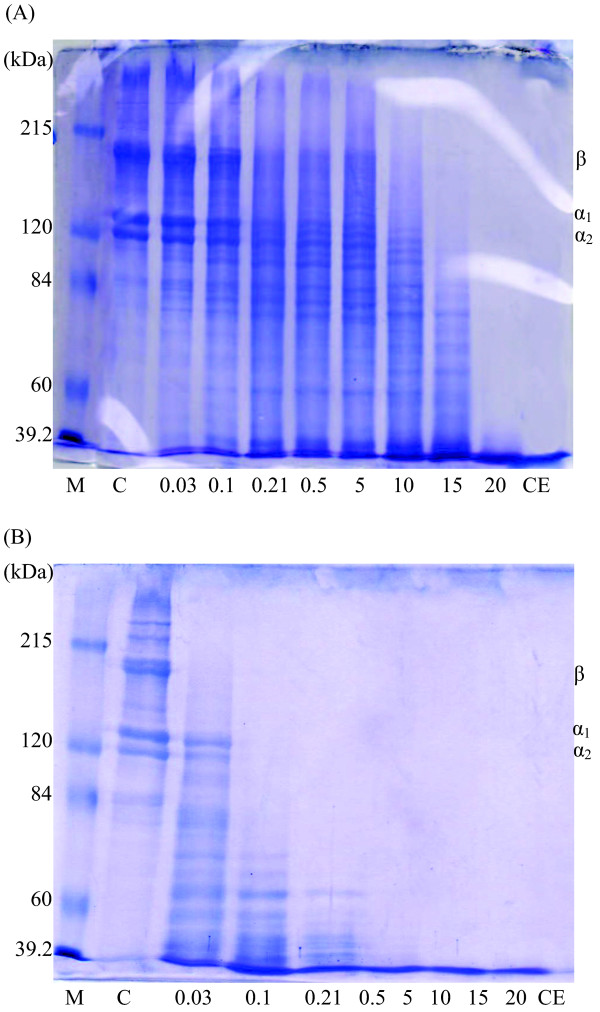
Peptide mapping of gelatin treated with the enzyme fraction (top phase) from the second cycle ATPS 15% PEG2000-15% sodium citrate containing 70% (w/w) crude enzyme extract with 10% additional sodium citrate (6A) and Flavourzyme (6B) [4% stacking gel and 7.5% separating gel SDS-PAGE]; M: molecular weight protein makers; C: control; 0.03, 0.1, 0.21, 0.50, 5, 10, 15, and 20 units: the unit of enzyme (initial enzyme 31.70 units); CE: crude enzyme extract.

The protein patterns of gelatin from farmed giant catfish skin treated with extracted alkaline protease compared to commercial Flavourzyme (initial activity 31.70 units) are depicted in Figure 6. For both enzymes, hydrolytic patterns of gelatin were clearly observed when the unit of enzyme increased indicating that the amount of very small molecules increased with unit of enzyme used. Disappearances of major components (β, α_1_ and α_2_) of the gelatin were observed when the addition level was 10 units and 0.21 units by extracted alkaline protease and Flavourzyme, respectively. Complete hydrolysis was found at the enzyme concentration of 20 units and 0.5 units for extracted alkaline protease and Flavourzyme, respectively. At the same unit of enzyme activity, gelatin treated with Flavourzyme showed more protein degradation than that treated with the extracted alkaline protease from farmed giant catfish viscera, indicating a higher specific activity of Flavouzyme towards gelatin, compared with extracted enzyme. Due to their digestive functions, viscera usually contain mechanisms with diverse functions, such as pepsin, elastase, trypsin, and chymotrypsin. Apart from pepsin, most digestive enzymes from viscera are endopeptidase and active at alkaline pH, over a temperatures range of 35 to 60°C [7]. Thus, under the hydrolysis conditions applied in this study (pH 8.5, 37°C), the activities of the alkaline protease of giant catfish viscera might not reach an optimum condition. Besides, it has been proved that in spite of the high amount of endogenous enzymes in viscera, the use of both endogenous and exogenous enzymes like Flavourzyme enhances the hydrolysis of proteins [28]. Endoproteases work by cleaving peptide bonds in the interior of polypeptide chains, whereas exopeptidases cleave off amino acids one at a time from the end of polypeptide chains [29]. The result showed that proteins or peptides were too small to be detected under the analytical SDS-PAGE using 7.5% separating gel electrophoresis conditions. From the peptide mapping, demonstrating that the alkaline protease extracted from farmed giant catfish viscera can further be used for gelatin hydrolysate production. From the previous study of Khantaphant et al. [27], gelatin treated with proteases of pyloric caeca extracted from brownstripe red snapper showed that no β-chain and α-chain were remained in hydrolysates with 5-10% degree of hydrolysis**.** Other studies [27,30] reported the success in using proteases from fish viscera in production of gelatin hydrolysate with antioxidative and angiotensin converting enzyme (ACE) inhibitory activities.

## Conclusions

ATPS with 15% PEG2000-15% sodium citrate, pH 8.5, containing 70% (w/w) crude enzyme extract provided the best enzyme recovery and purity. The pH and NaCl concentration had no effect on partitioning of the target enzyme. Addition of more salt content could improve the enzyme recovery in the second ATPS. Based on the protein degradation of gelatin, the alkaline protease from farmed giant catfish viscera can further be used in production of protein hydrolysate. ATPS could be potentially improved and applied as a commercially viable process for purification of proteases or other enzymes from any fish species because the method is rapid, efficient, cost-effective, and easy to scale up for industrial production. Especially, the repetitive ATPS exhibited exceedingly increase in purity and recovery. As a result, extracted alkaline protease from fish viscera, with a high purification factor and recovery, could be an excellent choice in application of food, detergent, biotechnology, and pharmacology industries.

## Methods

### Chemicals and reagents

Polyethylene glycol (PEG) 2000, sodium dodecyl sulfate (SDS) and bovine serum albumin (BSA) were obtained from Fluka (Buchs, Switzerland). Flavourzyme (protease, from *Aspergillus oryzae* ≥500 U/mg; code P6110), Beta-mercaptoethanol (βME) and Coomassie Brilliant Blue G-250 were purchased from Sigma Chemical Co. (St. Louis, MO, USA). *N*,*N*,*N*,*N*-tetramethyl ethylene diamine (TEMED) was purchased from Bio-Rad Laboratories (Hercules, CA, USA). Sodium citrate (Na_3_C_6_H_5_O_7_) and sodium chloride (NaCl) derived from Univar (Ajax Finechem, Australia). Trichloroacetic acid (TCA), hydrochloric acid, sodium hydroxide, tris-(hydroxymethyl)-aminomethane and other chemicals with analytical grade were obtained from Merck (Darmstadt, Germany).

### Gelatin preparation

Gelatin will be extracted from prepared fish skin of farmed giant catfish (*Pangasianodon gigas*) as described in Sai-Ut et al. [21]. Washed skin will be soaked in 0.2 mol/L NaOH (1:10 of a skin (g) to solution (mL) ratio) at 4±1°C for 2 h with a continuous gentle stirring. Alkaline-treated skin will be then washed with tap water until pH<7.5 of washed water will be obtained. The alkaline-treated skins will be soaked in 0.05 mol/l acetic acid with a skin to solution ratio of 1:10 (w/v) for 3 h at room temperature (25±1°C). Acid-treated skin will be washed as previously described. The swollen fish skin will be soaked in distilled water with a skin/water ratio of 1:10 (w/v) at 45±1°C for 12 h with continuous stirring to extract the gelatin. The mixture will be then filtered using two layers of cheesecloth. The resultant filtrate will be freeze dried and the dry matter from freeze-dried process was ground and referred to as “gelatin powder”.

### Preparation of crude enzyme extract

Viscera of farmed giant catfish were obtained from Charun Farm, Chiang Rai, Thailand. Those samples were packed in the polyethylene bag, kept in ice and transported to the Food Technology Laboratory, Mae Fah Luang University, Chiang Rai, Thailand, within 30 min. Pooled viscera were immediately frozen and stored at −20°C until used.

Frozen viscera were thawed using running tap water (26-28°C) until the core temperature reached (−2) ± 2°C. The sample was cut into small pieces and homogenized for 2 min with extraction buffer (10 mM Tris–HCl pH 8.0, containing 10 mM CaCl_2_) in the ratio of 1:5 (w/v). The mixture was centrifuged at 10,000*×g* for 10 min at 4°C. The pellet was discarded and the supernatant was collected and referred to as “crude enzyme extract” (CE). Protein concentration and protease activity in CE were measured.

### Protease partitioning by ATPS

#### Effect of crude enzyme extracts on protease partitioning

ATPS were prepared in 15 ml graduated centrifuge tubes by weighing. The ATPS containing different amounts of CE 20, 30, 40, 50, 60 and 70% (w/w) was added into the system consisting of 15% PEG2000-15% sodium citrate that provided the best alkaline protease recovery from the study of Vannabun and Rawdkuen [14,15]. Distilled water was used to adjust the system to obtain the final weight of 10 g. The mixtures were mixed thoroughly for 15 min using a Vortex mixer before pH measurement. Phase separation was achieved by centrifuging at 4000*×g* for 10 min at 4°C. The top phase was carefully separated using a Pasteur pipette. Volumes of the separated top and bottom phases were measured and recorded, after that both phases were put aside for the protease assay and total protein determination. Separation parameters; enzyme partition coefficient (K_E_), volume ratio (V_R_) purification fold (PF) and protease recovery (%) were also calculated according to Nalinanon et al. [1]. From the protease recovery, the protease obtained from the ATPS fraction that rendered the maximal recovery was chosen for further study.

#### Effect of pH on the protease partitioning

Based on the protease recovery, the ATPS containing the highest recovery from the first step was chosen for optimization. The original pH of the system was measured and then adjusted to 6.0, 7.0, 8.0 and 9.0, 11.0 by 1 M HCl or 1 M NaOH. ATPS for this step were done and determined as previously described. ATPS were prepared and monitored as previously described.

#### Effect of NaCl on the protease partitioning

The selected system showed the highest recovery from the previous step was chosen for study the effect of NaCl on protease partitioning. The powdered NaCl was directly dissolved into the systems, to achieve the final concentrations of 1, 3, 5 and 7% (w/w). ATPS for this step were prepared and monitored as previously described.

#### Repetitive ATPS for protease recovery

In the case of repetitive operation, the top phase from the system of 15% PEG 2000-15% sodium citrate, containing 70% (w/w) CE which showed the highest recovery was used as the starting material for the second ATPS by mixing with additional sodium citrate to obtain the final concentrations of 10, 15, and 20% (w/w). After the complete phase separation, the phases were collected as previously mentioned. The upper phases from the second ATPS were subjected to protein content and protease activity analysis.

All experiments were run in triplicate. The ATPS rendering the most effective protease partitioning was chosen. Phase giving the highest enzyme recovery was chosen for gelatin hydrolysis study.

### Characterization of alkaline protease

#### Proteolytic activity and protein content

Proteolytic activity was determined by using caseinolytic activity assay according to the method of Rawdkuen et al. [15] with slight modification. A volume of 0.5 ml of the enzyme sample was mixed with 0.5 ml of 1% (w/v) casein in 0.10 M of Tris–HCl (pH 8.0). The reaction was initiated by incubating the mixture at 37°C for 10 min. The reaction was stopped by adding 0.5 ml of 5% (w/v) TCA. After 5 min of centrifugation at 10,000×g, the absorption of the soluble peptides in the supernatant was measured at 280 nm. One caseinolytic activity unit is defined as the amount of enzyme needed to produce an increment of 0.01 absorbance unit per minute at the assayed condition.

Bradford method [31] was used for determination of protein concentration and bovine serum albumin (BSA) was used as a standard.

#### Gel electrophoresis

The protein pattern of the extracted protease was evaluated using SDS-PAGE according to the method of Laemmli [32]. The protein solutions were mixed at a 1:1 (v/v) ratio with the sample buffer (0.125 M Tris–HCl, pH 6.8, 4% SDS, 20% glycerol). The samples (8 μg and 2 μg for protein and activity staining, respectively) were loaded onto a 4% stacking gel and a 15% separating gel. The samples were subjected to a constant current of 15 mA/gel. After electrophoresis, the gel was stained overnight with a solution of 0.1% (w/v) Coomassie Brilliant Blue R-250 in 45% (v/v) methanol and 10 % (v/v) acetic acid. The gels were then destained with 50% (v/v) methanol and 7.5% (v/v) acetic acid for 30 min, followed by 5% (v/v) methanol and 7.5% (v/v) acetic acid for 15 min before being washed and dried.

#### Zymography

The protein band separated on SDS-PAGE was verified for proteolytic activity by using casein substrate gel electrophoresis according to the method of Garcia-Carreno et al. [33]. The gel was immersed in 50 ml of 2% (w/v) casein in 100 mM of a Tris–HCl buffer at pH 8.0, followed by constant agitation at 4°C for 45 min. The reaction was initiated by incubating the gel at 37°C for 15 min. The treated gel was then stained and de-stained as described above. The development of a clear band on the dark background indicated the caseinolytic activity of the proteases from the viscera of the farmed giant catfish. The gels were fixed and stained with Coomassie Blue R-250. Development of clear zones on blue background indicated proteolytic activity.

### Gelatin hydrolysis

Extracted alkaline protease from the ATPS providing the highest recovery (15% PEG2000-15% sodium citrate, pH system 8.5 containing 70% (w/w) CE with 10% additional sodium citrate) and Flavourzyme (initial activity 31.70 units) were used to prepare gelatin hydrolysate. The reaction was started by incubating the gelatin solution (2 mg/ml) with enzymes at different concentrations (0.03, 0.10, 0.21, 0.50, 5.00, 10.00, 15.00, and 20.00 units) at 37°C for 10 min and then terminated by submerging the mixture in boiling water for 3 min. Pattern of proteins generated was determined by SDS-PAGE using 7.5% separating gel and 4% stacking gel as mentioned above.

## Competing interests

The authors declare that they have no competing interests.

## Authors’ contributions

SK: carried out the whole experimental works, manuscript drafting and correcting. SB: participated in giving advises and final approval of the manuscript. TCL: participated in giving advises and final approval of the manuscript. OMA: participated in giving advises and final approval of the manuscript. SR: carried out the experimental design, data interpretation, manuscript revising and corresponding. All authors read and approved the final manuscript.
